# Prospective association between objective measures of childhood motor coordination and sedentary behaviour in adolescence and adulthood

**DOI:** 10.1186/s12966-015-0236-y

**Published:** 2015-06-10

**Authors:** Lee Smith, Abigail Fisher, Mark Hamer

**Affiliations:** Department of Epidemiology and Public Health, Health Behaviour Research Centre, University College London, England, WC1E 6BT UK; National Centre Sport & Exercise Medicine, School of Sport, Exercise and Health Sciences, Loughborough University, England, WC1E 6BT UK; Department of Epidemiology and Public Health, Physical Activity Research Group, University College London, England, WC1E 6BT UK

**Keywords:** Motor coordination, Physical activity, Sedentary, Birth cohort

## Abstract

**Background:**

Higher levels of gross motor coordination are positively associated with physical activity in childhood, but little is known about how they relate to sedentary behaviour. The aim of this study was to investigate the longitudinal association between gross motor coordination at childhood and sedentary behaviour in adolescence and adulthood.

**Methods:**

Data were from the 1970 British Cohort Study (the age 10, 16, and 42-year surveys). At age 10 the participant’s mother provided information on how often participants watched TV and played sports and a health visitor administered several tests to assess gross motor coordination. At aged 16 and 42-years participants reported their daily screen and TV time, respectively, and physical activity status. We examined associations between gross motor coordination at age 10 with sedentary behaviour and physical activity at age 16 and 42, using logistic regression.

**Results:**

In multivariable models, higher levels of gross motor coordination were associated with lower odds of high screen time (*n* = 3073; OR 0.79, 95 % CI 0.64, 0.98) at 16-years although no associations with physical activity were observed (OR 1.16, 95 % CI 0.93, 1.44). Similar associations were observed with TV time in adulthood when participants were aged 42, and in addition high gross motor coordination was also associated with physical activity participation (*n* = 4879; OR 1.18, 95 % CI 1.02, 1.36).

**Conclusions:**

Intervention efforts to increase physical activity participation and reduce sedentary behaviour over the life course may be best targeted towards children with low gross motor coordination.

**Electronic supplementary material:**

The online version of this article (doi:10.1186/s12966-015-0236-y) contains supplementary material, which is available to authorized users.

## Background

Population levels of physical activity in westernised countries are low and sedentary time – defined as any waking behaviour characterised by energy expenditure below 1.5 metabolic equivalents while in a sitting or reclined posture – high [1]. Regular participation in physical activity aids in the prevention against non-communicable disease risk factors and a high level of sitting (ie, sedentary time) is detrimental to adult health, independently of physical activity levels [2, 3]. Recent UK physical activity guidelines also include recommendations to reduce sedentary activities [4].

To date, interventions to increase population levels of physical activity and reduce sedentary behaviour have demonstrated limited success, reflected in the current low population levels of physical activity and high sedentary time [1] possibly because they are not targeted enough. Interventions may be more successful if we can identify correlates of physical activity and sedentary behaviours and groups at risk of low levels of physical activity. A large body of literature exists on correlates of physical activity in adulthood [5]. Physical activity participation is consistently higher in men than in women, inversely associated with age, and lower in participants from more socially disadvantaged backgrounds. Literature is also beginning to emerge on correlates of specific sedentary behaviours. A systematic review [5] showed higher levels of TV viewing in adults to be associated with lower education, older age, unemployment or lower working hours, and higher body mass index (BMI), independent of sex. Mixed evidence was found for associations between TV viewing and marital status, income and ethnicity. Higher TV viewing was also found to be associated with lower leisure time physical activity and having a TV in the bedroom. However, the majority of studies in these areas are of a cross-sectional nature.

There is a growing body of literature on the relationship between gross motor coordination and participation in physical activity in young people, although there are limited longitudinal studies that track into adulthood. Furthermore, most studies have focused on physical activity and not on sedentary behaviour. The majority of studies carried out in this area have found a positive association between higher gross motor coordination and physical activity participation (eg, see reviews Lubans et al. [6] and Holfelder et al. [7]). For example, Wrotniak et al. [8] found that children’s motor proficiency was positively associated with activity counts and percentage of time in moderate and moderate-to-vigorous intensity activity and inversely related to percentage of time in sedentary activity. It is possible that children with poor gross motor coordination may not find physical activity to be enjoyable as it may be more challenging for them compared to those who have good gross motor coordination. It is also plausible to assume that for children with poor gross motor coordination sedentary activities (i.e., TV viewing and computing gaming) may be more enjoyable options. Vice versa, excessive sedentary time may inhibit development of gross motor coordination [9]. This may be problematic as sedentary behaviour and physical activity have been shown to track from childhood to adulthood [10, 11]. A recent study [12] in a birth cohort found that hand control/ coordination problems and low sports aptitude was associated with inactivity (not meeting physical activity guidelines) in adulthood. However, no study has investigated whether components of motor coordination at childhood predict sedentary time in adulthood. The present analysis aims to investigate the longitudinal association between motor coordination at childhood and physical activity and sedentary participation at adulthood.

## Methods

The 1970 British Cohort Study (BCS70) follows the lives of 17,284 people born in England, Scotland and Wales in a single week of 1970. The present analyses incorporated data from the age 10, 16 and age 42 surveys. At the age 10 survey, conducted in 1980/81, parents provided informed consent and were interviewed about the child’s home background, social experience, a number of factors concerning the experiences of the child and the family. The information was gathered through a structured interview with the mother of the child, or if she was not available, with someone who had knowledge on the child’s health and development. The age 16 survey was conducted in 1986 and importantly, for the first time, contained a participant self-completion section on health related behaviour. The age 42 survey was conducted in 2012/13 and comprised of a 60 min face-to-face computer-assisted-personal-interview, which included a vocabulary task and a self-completion section. At the age 10 survey 14,874 cohort members participated; 6898 (46.4 %) completed the self-completion module at age 16; and 9,842 (66.2 %) took part in the age 42 survey. The lower response at 16 arose because of a teachers’ strike that resulted in many participants not receiving their questionnaires. Participants provided informed consent and all data collection on BCS70 has received full ethical approval (London Central REC).

### Gross motor co-ordination variables at Age 10

A health visitor administered several tests to assess gross motor coordination.

#### Throwing test

The child was given two to three attempts to test if they could throw a tennis ball up in the air and catch it. For children that were successful a second test involved throwing the ball and clapping hands together once before catching. The procedure was repeated increasing the number of claps until the child failed two successive attempts. These tests were then repeated using only one hand to catch the ball. One point was assigned if the child achieved at least 3 claps whilst catching the ball with one hand.

#### Standing on one leg

The child was asked to stand on their right leg with the left foot positioned on the right knee and maintain this posture for 30 s. One point was assigned if the child remained balanced on one leg without moving their left foot.

The child was then asked to stand on their right leg with the left foot positioned on the right knee and maintain this posture for 30 s. One point was assigned if the child remained balanced on one leg without moving their left hand.

#### Walking backwards

A four metre line was marked out with chalk. The child was asked to walk backwards placing one foot behind the other (toe-to-heel). The child was allowed to have two practice attempts for 5 steps. The child was then asked to walk backwards for 20 steps. The number of steps was recorded before making an error, construed as ceasing to make toe to heel contact, deviating from line, or removing hands from hips. One point was assigned if the child achieved the 20 steps.

Children were split into three categories reflecting ‘Low’ (0 or 1 point), ‘Medium’ (2 points), ‘High’ (3 or 4 points) gross motor coordination.

### Sedentary time and physical activity at follow up (Age 16 and 42)

To measure habitual physical activity at age 16 respondents were provided with a list of 34 sports and physical activities (see Additional file 1: Table S1) and asked which of them they played (in school and during leisure time) when they were in season during the past year (at least once a week; once a month; not at all). Respondents were asked separate questions about how long they spent in three types of sedentary activities (TV, video films, computer games) after school yesterday (not at all; less than 1 h; > 1 h; >2 h; >3 h; >4 h; >5 h). Responses were substituted with dummy variables (ranging from 0 – 6) and then summed across the 3 questions in order to estimate total screen time.

At age 42 respondents reported how many hours they spent watching TV per day (none/0 ≤ 1/ 1 < 3/ 3 < 5/ ≥5) and frequency of participation in 15 types of physical activities and sports (see Additional file 2: Table S2) (every day/5–6 times a week/2-3 times a week/ once a week/2-3 a month/less often/not in last 12 months).

#### Covariables

At age 10, the cohort member’s mother provided information regarding how often their child watched TV and played sports (categorised as: never/sometimes/often). The health visitor recorded height and body mass for the calculation of body mass index (BMI). Parents provided information on their occupation, which was categorised using the 1970 and 1980 Office of Population Censuses and Surveys Classification of Occupations (Managerial/Professional/Intermediate [skilled & non-skilled]/Routine and manual), smoking habits, and also provided self-reported weight and height, from which BMI was calculated.

#### Statistical analysis

We examined associations between childhood motor-coordination at aged 10 with sedentary behaviour and physical activity at age 16 and 42 using logistic regression models. Screen time at age 16 and TV time at age 42 was categorised into binary variables with the highest group roughly reflecting the upper third of the distribution (<3 h per d or ≥ 3 h/day). Physical activity was also treated as a binary outcome variable; at age 16, physical activity was categorised as (never/infrequent or at least once a week); at age 42 it was categorised as “at least 2–3 times a week” as adults participating in activity at least 2–3 times a week are likely to approach the minimum physical activity recommendation of 150 mins/week. We calculated odds ratios (OR) and 95 % confidence intervals (CI) for the risk of high screen (or TV) time/ physical activity participation at age 16 and 42. Initially we performed analysis adjusted for sex. We then further adjusted the models for child BMI age 10, child TV viewing age 10, child sports participation age 10, father’s occupation, father’s BMI, and mother’s smoking habit. All analyses were conducted using SPSS version 22.

## Results

At age 10 the gross motor coordination tests were completed in 10,831 children. In comparison to children who completed the tests, those that did not (*n* = 4043) were less likely to be girls (49.7 vs 43.9 %, *p* < 0.001), more likely to have a mother who smoked (40.8 vs. 43.0 %, *p* = 0.04), but were similar in other characteristics such as father’s occupational class (% routine/manual; 17.7 vs. 16.0 %, *p* = 0.08), and TV viewing habits at age 10 (% often viewing TV; 79.3 vs. 79.0 %, *p* = 0.70). A summary of gross motor coordination test performance is presented in Table 1. In general, higher task performance was observed in girls; in participants with a father in professional and managerial occupations; in those with non-smoking mothers; and in participants that played sport. The descriptive characteristics of the sample and the association of the covariables with screen time and physical activity at age 16 are presented in Additional file 3: Table S3.

**Table 1:** Gross motor co-ordination tests in BCS70 age 10

Test	% sample
Balancing on one leg for 30 s without moving foot	66.1
Balancing on one leg for 30 s without moving hand	76.1
Throwing and catching task with one hand, (# claps)	
0 – 1	16.7
2	36.5
3	32.2
4 or more	14.6
Walking backwards (# steps)	
0 – 10	21.4
11 – 19	28.3
20	50.3

At age 16 over a third (36.3 %) of respondents reported more than 3 h screen time in total after school. TV time accounted for the majority of total screen time at age 16, (91.1 % of the sample reported “not at all” playing computer games after school). In multivariable models, high gross motor coordination was associated with lower odds of high screen time (*n* = 3073; OR 0.79; 95 % CI 0.64 to 0.98) although no associations with physical activity were observed (*n* = 3073; OR 1.16; 95 % CI 0.93 to 1.44; Table 2). Similar associations were observed with TV time in adulthood when participants were aged 42 (*n* = 4879; OR 0.85; 95 % CI 0.72 to 0.99), and in addition high gross motor coordination was also associated with physical activity participation (*n* = 4879; OR 1.18; 95 % CI 1.02 to 1.36; Table 3).

**Table 2:** Association between gross motor-coordination age 10 and physical activity/sedentary behaviour age 16 (*n* = 3073)

Motor co-ordination category	After school screen time (>3 h/day)		Physical activity participation (>1/week)	
	Model 1	Model 2	Model 1	Model 2
	OR (95 % CI)	OR (95 % CI)	OR (95 % CI)	OR (95 % CI)
Low (*n* = 637)	1.0 (Ref)	1.0	1.0 (Ref)	1.0
Medium (*n* = 792)	0.76 (0.60, 0.96) z	0.80 (0.63, 1.02)	0.98 (0.77, 1.25)	0.98 (0.78, 1.25)
High (*n* = 1644)	0.74 (0.60, 0.92)	0.79 (0.64, 0.98)	1.20 (0.97, 1.48)	1.16 (0.93, 1.44)

Model 1 adjusted for sex

Model 2 adjusted for sex, child BMI age 10, child TV viewing age 10 (hardly ever/often), child sports participation age 10 (hardly ever/often), father occupational class, father BMI, parents smoking habit

**Table 3:** Association between gross motor-coordination age 10 and physical activity/sedentary behaviour age 42 (*n* = 4879)

Motor co-ordination category	TV viewing (>3 h/day)		Physical activity participation (>1/week)	
	Model 1	Model 2	Model 1	Model 2
	OR (95 % CI)	OR (95 % CI)	OR (95 % CI)	OR (95 % CI)
Low (*n* = 1125)	1.0 (Ref)	1.0	1.0 (Ref)	1.0
Medium (*n* = 1255)	0.87 (0.72, 1.04)	0.90 (0.75, 1.09)	1.11 (0.94, 1.30)	1.08 (0.92, 1.27)
High (*n* = 2499)	0.80 (0.68, 0.94)	0.85 (0.72, 0.99)	1.22 (1.06, 1.41)	1.18 (1.02, 1.36)

Model 1 adjusted for sex

Model 2 adjusted for sex, child BMI age 10, child TV viewing age 10 (hardly ever/often), child sports participation age 10 (hardly ever/often), father occupational class, father BMI, parents smoking habit

## Discussion

The present study found that gross motor coordination at ten years old was inversely associated with screen time at 16 years and TV time at 42 years of age. Gross motor coordination was only associated with participation in physical activity at age 42. Prior cross-sectional studies on childhood gross motor coordination have not been able to tease apart if poor gross motor coordination leads to more sedentary time or the converse. This is the first study to use a longitudinal design in a population based sample to examine associations between childhood gross motor coordination and sedentary behaviour in adolescence and adulthood. Thus, our data support the notion that poor gross motor coordination leads to sedentary behaviour. Our data support the literature investigating associations between components of gross motor coordination and physical activity in childhood [10, 11]; and are consistent with the only other study investigating childhood correlates of adult physical activity behaviour, which also found childhood motor coordination (measured by hand control/ coordination problems) to predict physical activity participation in adulthood [12]. As previously hypothesised, children with poor gross motor coordination may find physical activity less enjoyable as it may be more challenging for them, and thus may prefer more passive sedentary activities (ie, TV viewing and computing gaming). However, no current research exists to support this hypothesis. Our data do not allow us to test these explanations, or indeed probe for any mechanism for the observed effect, thus further research is warranted.

The present analysis found no association between childhood gross motor coordination and physical activity at 16 years. The lack of association found between gross motor-coordination at childhood and physical activity participation at 16 years may be due to the far cruder nature of the physical activity responses that prevented us from generating detailed data on activity volume.

Physical activity promotion and sedentary reduction interventions may wish to target children who have lower gross motor coordination as these interventions may encourage physical activity participation, and reduce their sedentary behaviour, throughout the life course; exposure to cardiometabolic risk factors throughout the life course may present the greatest health risks. Such interventions may not necessarily target sport *per se*, but focus on other domains of physical activity that do not require high levels of gross motor coordination, such as active travel (ie, walking or cycling). In a recent review on active travel [13], almost all studies reported an increase in the percentage of active travel to school following an intervention; however, the degree of change varied widely (3 % to 64 %). Interventions may also wish to focus on active outdoor play, as this may displace sedentary behaviour on evenings and weekends with more active recreational alternatives, in addition to promoting gross motor coordination. Such an intervention may target the level of independent mobility granted to a child. Studies have found associations between higher levels of independent behaviour and both higher levels of physical activity (including active travel) and time spent outdoors after school [13–15].

The present study has a number of limitations. We cannot exclude the possibility of residual confounding. For example, gross motor coordination may correlate with other biological risk factors influencing physical activity behaviour. Inconsistencies between parents (age 10 surveys) and participants (age 42 surveys) may have introduced bias into the present analyses. Nevertheless, using a birth cohort design is more advantageous than using retrospective recall in adulthood. At age 42 years the measures of sedentary behaviour was restricted to TV viewing time and therefore may have not captured total sedentary time. Owing to its passive nature and high prevalence, TV viewing may be difficult to recall accurately. Nevertheless, TV viewing at age 42 in the present study is broadly comparable with data on TV viewing from the Health Survey for England (http://www.hscic.gov.uk/catalogue/PUB00430/heal-surv-phys-acti-fitn-eng-2008-rep-v2.pdf), which is a representative sample of the general English population. Moreover sedentary time questions that focus on TV viewing have demonstrated the strongest reliability and validity among non-occupational sedentary behaviour questions [16].

The battery of tests to investigate gross motor coordination at baseline has not been compared against contemporary tests. Although more recognised measures of motor control now exist, it is important to highlight that gross motor coordination was measured over 30 years ago when such measures were not available. A clear strength of this study is its prospective design and 32 year follow-up in a population-based sample of English, Scottish and Welsh adults. A further strength is the inclusion of objective tests of motor coordination at childhood as a potential correlate of physical activity/sedentary behaviour in adulthood.

## Conclusions

This is the first study to use a longitudinal design to examine associations between childhood gross motor coordination and sedentary behaviour in adolescence and adulthood. The level of gross motor coordination during childhood was associated with physical activity participation and sedentary behaviour in adulthood. Intervention efforts to increase physical activity participation and reduce sedentary behaviour over the life course may be best targeted towards children with low gross motor coordination.

## Additional files

Additional file 1: Table S1. Individual sports/physical activity included at age 16. Additional file 2: Table S2. Individual sports/physical activity included at age 42. Additional file 3: Table S3: Association of baseline covariables with screen time and physical activity at age 16.
