# Supplementary material for: Prospective association between objective measures of childhood motor coordination and sedentary behaviour in adolescence and adulthood
Source: Int J Behav Nutr Phys Act. 2015 Jun 10;12:75. doi: 10.1186/s12966-015-0236-y (PMC4464137; doi:10.1186/s12966-015-0236-y)
Supplement: Additional file 1: Table S1. — Individual sports/physical activity included at age 16. [file 12966_2015_236_MOESM1_ESM.docx]

**Additional file 1: Table S1.** Individual sports/physical activity included at age 16

| Baseball |
| --- |
| Basketball |
| Cricket |
| Football |
| Hockey |
| Netball |
| Rounders |
| Rugby |
| Volleyball |
| Aerobics |
| Track/field athletics |
| Badminton |
| Canoeing |
| Cross country running |
| Cycling |
| Dancing |
| Gymnastics |
| Horse riding |
| Jogging |
| fitness |
| Motor cycling |
| Roller/ice skating |
| Rowing |
| Sailing |
| Climbing |
| Skiing |
| Squash |
| Swimming |
| Table tennis |
| Tennis |
| Walking |
| Water skiing |
| Weight training |
| Wind surfing |
