# Supplementary material for: Prospective association between objective measures of childhood motor coordination and sedentary behaviour in adolescence and adulthood
Source: Int J Behav Nutr Phys Act. 2015 Jun 10;12:75. doi: 10.1186/s12966-015-0236-y (PMC4464137; doi:10.1186/s12966-015-0236-y)
Supplement: Additional file 2: Table S2. — Individual sports/physical activity included at age 42. [file 12966_2015_236_MOESM2_ESM.docx]

**Additional file 2: Table S2.** Individual sports/physical activity included at age 42

| Health, fitness, gym, conditioning activities |
| --- |
| Rambling / walking for pleasure |
| Swimming or diving |
| Cycling, BMX or mountain biking |
| Jogging, cross-country, road-running |
| Raquet sports |
| Team sports |
| Dancing |
| Yoga/ pilates |
| Golf |
| Martial arts, boxing, wrestling |
| Water sports |
| Horse riding |
| Skiing |
| Other sporting activity |

**Table S2.** Association between sports/outdoor play (age 10) and meeting physical activity guidelines (30 minutes exercise on at least 5 days a week) at age 42 (n=6,458).

|  | **Odds ratio (95% CI) for meeting PA guideline age 42** | | |
| --- | --- | --- | --- |
|  | Univariate model | Model 1† | Model 2‡ |
| ***Active outdoor play at age 10***  *Never/ sometimes*  *Often* *(daily)* | Ref  1.00 (0.89 – 1.13) | Ref  0.97 (0.86 – 1.10) | Ref  0.96 (0.85 – 1.09) |
| ***Playing sports at age 10***  *Never/ sometimes*  *Often* *(daily)* | Ref  1.17 (1.04 – 1.32) | Ref  1.03 (0.91 – 1.17) | Ref  1.03 (0.91 – 1.17) |

†Model 1 adjusted for sex, sports or active play age 10, fathers occupation, child BMI, and father BMI.

‡Model 2 additionally adjusted for: self-rated health age 42 (excellent; very good; good; fair; poor); assessment of own weight at age 42 (about right; underweight; overweight; very overweight), participant’s education (higher education; A-levels; GCSEs/O-levels; no education).
