# Supplementary material for: Prospective association between objective measures of childhood motor coordination and sedentary behaviour in adolescence and adulthood
Source: Int J Behav Nutr Phys Act. 2015 Jun 10;12:75. doi: 10.1186/s12966-015-0236-y (PMC4464137; doi:10.1186/s12966-015-0236-y)
Supplement: Additional file 3: Table S3: — Association of baseline covariables with screen time and physical activity at age 16. [file 12966_2015_236_MOESM3_ESM.docx]

**Additional file 3: Table S3**. Association of baseline covariables with screen time and physical activity at age 16.

| Variable at baseline | **Total sample**  (n=3073) | **After school screen time** | | **Physical activity** | |
| --- | --- | --- | --- | --- | --- |
|  |  | < 3hrs  (n=1958) | >3 hrs  (n=1115) | None or < 1 a week  (n=667) | At least 1 per week  (2406) |
| Sex (%)  *Boys*  *Girls* | 43.3  56.7 | 58.3  67.8 | 41.7  32.2 | 20.7  22.5 | 79.3  77.5 |
| Child BMI age 10 (kg/m^2^) | 16.9 ± 2.1 | 16.9 ± 2.1 | 16.9 ± 2.1 | 16.7±2.0 | 17.0±2.1 |
| TV viewing age 10 (%)  *Hardly ever/sometimes*  *Often* | 21.7  78.3 | 72.7  61.1 | 27.3  38.9 | 22.2  21.6 | 77.8  78.4 |
| Sports age 10 (%)  *Hardly ever/sometimes*  *Often* | 46.9  53.1 | 64.4  62.6 | 35.6  37.4 | 25.5  18.4 | 74.5  81.6 |
| Father occupational class (%)  *Managerial*  *Professional*  *Intermediate*  *Routine and manual* | 8.8  29.2  49.8  12.2 | 78.3  71.0  58.9  54.7 | 21.7  29.0  41.1  45.3 | 28.3  21.1  20.9  21.2 | 71.7  78.9  79.1  78.8 |
| Father BMI (kg/m^2^) | 24.3 ± 2.9 | 24.3±2.9 | 24.4±3.0 | 24.1±3.0 | 24.4±2.9 |
| Parental smoking (%)  *Yes*  *No* | 33.4  64.7 | 61.6  64.9 | 38.4  35.1 | 19.7  22.5 | 80.3  77.5 |
